# Mining Milk for Factors which Increase the Adherence of *Bifidobacterium longum* subsp. *infantis* to Intestinal Cells

**DOI:** 10.3390/foods7120196

**Published:** 2018-12-03

**Authors:** Erinn M. Quinn, Helen Slattery, Aoife P. Thompson, Michelle Kilcoyne, Lokesh Joshi, Rita M. Hickey

**Affiliations:** 1 Teagasc Food Research Centre, Moorepark, Fermoy, Co. Cork P61 C996, Ireland; erinnquinn12@hotmail.com (E.M.Q.); helen.slattery@teagasc.ie (H.S.); aoifethompson85@gmail.com (A.P.T.); 2 Advanced Glycoscience Research Cluster, National Centre for Biomedical Engineering Science, National University of Ireland Galway, Galway H91 TK33, Ireland; michelle.kilcoyne@nuigalway.ie (M.K.); lokesh.joshi@nuigalway.ie (L.J.)

**Keywords:** *Bifidobacterium*, adhesion, milk oligosaccharides, milk powders, HT-29 cells

## Abstract

Bifidobacteria play a vital role in human nutrition and health by shaping and maintaining the gut ecosystem. In order to exert a beneficial effect, a sufficient population of bifidobacteria must colonise the host. In this study, we developed a miniaturised high-throughput in vitro assay for assessing the colonising ability of bacterial strains in human cells. We also investigated a variety of components isolated from different milk sources for their ability to increase the adherence of *Bifidobacterium longum* subsp. *infantis* ATCC 15697, a common member of the gastrointestinal microbiota of breastfed infants, to HT-29 cells. Both conventional and miniaturised colonisation assays were employed to examine the effect of 13 different milk-derived powders on bacterial adherence, including positive controls which had previously resulted in increased bifidobacterial adherence (human milk oligosaccharides and a combination of 3′- and 6′-sialylactose) to intestinal cells. Immunoglobulin G enriched from bovine whey and goat milk oligosaccharides resulted in increased adhesion (3.3- and 8.3-fold, respectively) of *B. infantis* to the intestinal cells and the miniaturised and conventional assays were found to yield comparable and reproducible results. This study highlights the potential of certain milk components to favourably modulate adhesion of bifidobacteria to human intestinal cells.

## 1. Introduction

Milk is a complex and complete source of bioactive molecules that help to protect the newborn against infectious diseases and promote development, while selectively enriching a beneficial gut microbiota. Therefore, milk influences infant nutrition and microbial nutrition, which in combination promote infant health. Analyses of the infant microbiome indicate that breast milk selects for a highly adapted intestinal microbiota, dominated by bifidobacteria [1]. Indeed, the most commonly detected species in breastfed infants include *Bifidobacterium longum*, *B. infantis*, *B. breve*, and *B. bifidum* [2]. Bifidobacteria are associated with a number of health-related benefits for the host including inhibiting the growth of pathogenic organisms, modulating mucosal barrier function and promoting appropriate immunological and inflammatory responses [3,4]. Based on these therapeutic effects, bifidobacteria are a popular choice as probiotics. In order to exert a beneficial effect, a sufficient population of bifidobacteria must colonise the host, and therefore, initially adhere to host cell components [5]. There are two approaches most traditionally employed to modulate the gut microbiota; delivery of live bacteria (probiotics) within a food source, or the use of specific prebiotics (inulin, fructo- and galactooligosaccharides), which are known to survive gastric transit and are fermented in the colon by beneficial bacteria, thus promoting their growth [6].

More recently, studies have suggested that components in human milk, such as oligosaccharides, may contribute not only to the selective growth of commensal bacteria but also to their specific adhesive ability. Gonzalez et al. [7] demonstrated that growth of *B. longum* in defatted human milk leads to the genetic up-regulation of putative type II glycoprotein binding fimbriae, which have been implicated in attachment and colonisation. Chichlowski et al. [8] demonstrated that the growth of *B. longum* subsp. *infantis* ATCC 15697 on human milk oligosaccharides (HMO) as the sole carbon source increased bacterial adherence to HT-29 intestinal cells. Kavanaugh et al. [9] showed that treatment of *B. longum* subsp. *infantis* ATCC 15697 with a mixture of the HMO, 3′- and 6′-sialyllactose (3′SL and 6′SL, respectively) substantially increased bacterial adhesion (up to 9.8-fold) to HT-29 cells. Moreover, transcriptomic analysis revealed that the increased adherence phenotype of the strain resulting from exposure to HMO is likely multi-faceted, involving transcription factors, chaperone proteins, adhesion-related proteins, and a glycoside hydrolase [9]. Garrido et al. [10] found that solute binding proteins produced by *B. longum* subsp. *infantis* ATCC 15697 had a binding affinity for mammalian carbohydrate structures including Lewis antigens, polylactosamines and globotriose (Gb3) and structures found in colonic mucins, suggesting that *B. infantis* may also interact with such structures on the epithelial cells.

Examining the effect of HMO structures on bifidobacterial colonisation in vivo may prove difficult as only certain structures are currently available and not the entire range of HMO found in human milk. Therefore, investigation of other milk sources for components with adhesion promoting capabilities may be an attractive alternative. For instance, domestic animal milk components including oligosaccharides may have biological functions similar to those of human milk. In addition, current methods for investigation of bacterial colonisation are time-consuming and require a high quantity of sample for testing. As a result, there is a need to develop high-throughput (HTP) techniques which can quickly define the colonising ability of bacterial strains to human cell lines and which require substantially lower sample quantities. This is particularly important as isolation yields of compounds from natural sources are often too low for investigation in such bioassays.

The aim of the current study was to investigate the changes in adhesion of *B. longum* subsp. *infantis* ATCC 15697, a model consumer of HMO in the infant gastrointestinal (GI) tract, to HT-29 cells following exposure to a panel of milk-derived components. We have also developed a miniaturised HTP method for screening bacterial interactions with cells and compared it with a conventional assay. In total, 13 different milk powders derived from a variety of sources were investigated for their ability to increase bacterial adherence to HT-29 cells, using both conventional and miniaturised colonisation assays. Free oligosaccharides and some of the major milk glycoproteins comprised the main components of these powders and their effects were compared with commercially available 3′- and 6′–sialyllactose and HMO, which were previously shown to increase bifidobacterial adherence to HT-29 cells.

## 2. Materials and Methods

### 2.1. Materials

The oligosaccharides 3′-sialylactose (3′-SL) and 6′-sialylactose (6′-SL) were purchased from Carbosynth Ltd. (Berkshire, UK). Beneo Orafti P95 (oligofructose) was kindly provided by Beneo Orafti (Oreye, Belgium)**.** Bovine β-casein (β-C) and β-lactoglobulin (β-L) were purchased from Merck (Darmstadt, Germany). Glycomacropeptide (GMP), with a maximum lactose content of 1% and containing approximately 8.5% sialic acid was kindly provided by Agropur Ingredients (Eden Prairie, MN, USA). Lyophilised Bovine Lactoferrin (LF) and lyophilised Immunoglobulin G enriched powder (IGEP) isolated from bovine whey were kindly provided by Upfront Chromatography (Copenhagen, Denmark). Lacprodan^®^ MFGM-10 from whey protein concentrate with a maximum lactose content of 3%, containing 2% sialic acid and 5% IgG was kindly provided by Arla Food Ingredients (Viby J, Denmark). Lacprodan^®^ PL-20 with a maximum lactose content of 10%, containing a minimum of 16% phospholipids was also kindly provided by Arla Food Ingredients. The human colonic adenocarcinoma HT-29 cell line was used as a model of the human intestinal epithelial layer and was purchased from the American Type Culture Collection (ATCC, Middlesex, UK).

### 2.2. Generation of Milk-Derived Powders

All powders used in the study and their sources are listed in Table 1. For human milk oligosaccharides (HMO), human milk was kindly donated by the Irvinestown Human Milk Bank (Co. Fermanagh, Ireland). For goat milk oligosaccharides (GMO), mature milk from goats was kindly donated by Ardsallagh Goat Farm (Carrigtwohill, County Cork). All samples were stored at −80 °C on arrival. To generate low molecular weight fractions, the milks were initially defatted by centrifugation at 4 °C, for 20 min at 3850× *g*. Caseins were then precipitated by adjusting the pH to 4.6, followed by centrifugation at 3850× *g* at 25 °C for 20 min. After neutralisation (by adjusting the pH to 6.7), large peptides and whey proteins were removed by ultrafiltration using a 5 kg/mol molecular weight cut off (MWCO) membrane (Millipore Helicon S10 Spiral Cartridge; Millipore). The permeates were freeze-dried and, to separate the lactose from the oligosacchrides, 100 mL of a 10% solution of each powder were applied to a BioGel P2 size exclusion column (92 × 5 cm; Bio-Rad Laboratories, Inc., Hercules, CA, USA) and eluted with deionised water at 3 mL/min. The 14 mL fractions collected were analysed for lactose, 3-SL and 6-SL using high pH anion exchange chromatography with pulsed amperometric detection (HPAEC-PAD) as detailed below, and peptide concentration was determined by the Bradford assay [11]. Peptide-free and low-trace lactose (<80 mg/L) fractions were pooled and freeze-dried to give an oligosaccharide-enriched fraction (referred to as HMO and GMO for human and goat milk, respectively).

For bovine milk oligosaccharides (BMO), mother liquor (Glanbia plc, Co., Kilkenny, Ireland) was treated as described above. Following this, fractions low in lactose (<30 mg/L) from a number of separate runs were pooled and freeze-dried to give a bovine oligosaccharide enriched powder which still contained residual peptides. A 5% filtered solution (100 mL) of this powder was applied to a Nucleosil C18 reverse phase column (250 × 8 mm) on a 2695 Waters Alliance HPLC system (Waters Corporation, 34 Maple St, Milford, MA, USA) using deionised water as the eluent at a flow rate of 2 mL/min, which resulted in the binding of peptides to the column. After the sample was applied, the column was washed with one column volume of water. The sample flow-through and column wash were eluted, collected and freeze-dried to give an oligosaccharide-enriched and peptide-free fraction referred to as BMO.

For buttermilk fractions (BF), milk from Holstein-Friesian cattle was obtained from the bulk tank located at the milking parlour at the Teagasc Food Research Centre, Moorepark (Fermoy, Co., Cork, Ireland). Isolation of the buttermilk fraction was performed as previously described [12]. In brief, cream and milk fat globules were separated from the milk using a FT15 disc bowl centrifuge (Armfield Ltd., Ringwood, UK) and the cream was stored at 4 °C for 12–24 h. After chilling, the cream was churned using a mixer to produce butter and buttermilk. The buttermilk was then passed through glass wool (Merck) to remove minute butter granules. Washed cream was produced as described previously [13,14]. Briefly, warm deionised water (37 °C) was added to the cream at a ratio of 1:10 and the mixture was added to the FT15 disc bowl centrifuge. This process was repeated twice, and the washed cream was then used to produce buttermilk as described above. A 5 kg/mol Viva-Flow 200 (Sartorius^®^, Göttingen, Germany) crossflow filtration system was used to separate lactose from the high molecular weight components leaving a sample enriched in glycoproteins and glycolipids. The sample was then lyophilised and the dry powder stored in a desiccator at room temperature. The final sample contained 0.223 mg/g of lactose which was quantified as previously described [15].

### 2.3. Milk Oligosaccharides Analysis

Oligosaccharide-enriched fractions were diluted in water and analysed in order to quantify levels of lactose, 3′-SL and 6′-SL using a Dionex ICS-3000 Series system (Dionex Corporation, Sunnyvale, CA, USA) equipped with an electrochemical detector. Samples were separated on a CarboPac PA100 column (250 × 4 mm) equipped with a guard column using the following gradient; 95% 100 mM NaOH (Eluent A) and 5% 100 mM NaOH with 500 mM NaAc (Eluent B) for 3 min, 88% eluent A and 12% eluent B for 10 min, and 50% eluent A and 50% eluent B for 17 min for a 30 min separation. The column was re-equilibrated for 15 min with 95% eluent A and 5% eluent B after each separation.

### 2.4. Bacterial Strains and Culture

Bacterial culture conditions were maintained as previously described [9]. *Bifidobacterium longum* subsp. *infantis* ATCC^®^ 15697™ (*B. infantis*) was obtained from the American Type Culture Collection (ATCC, Middlesex, UK). and the strain was stored in deMan Rogosa Sharpe (MRS) (Difco, Sparks, MD, USA) broth containing 50% glycerol at −80 °C. The strain was cultured twice in MRS media supplemented with L-cysteine (0.05% *w*/*v*) prior to use and was routinely grown overnight at 37 °C under anaerobic conditions generated using the Anaerocult A system (Merck, Dannstadt, Germany). For assays, *B. infantis* was incubated alone or in combination with each powder for 1 h and the bacteria were serially diluted and enumerated by spot plating on MRS agar to determine bacterial numbers. To determine the mid-exponential growth phase, the culture was monitored by measuring the absorbance at 600 nm (OD_600 nm_) at intervals during growth.

### 2.5. Mammalian Cell Culture

Typically, HT-29 cells were grown in McCoy′s 5A modified medium (Merck) supplemented with 10% fetal bovine serum (FBS), which were maintained in 75 cm^2^ tissue culture flasks and incubated at 37 °C in 5% CO_2_ in a humidified atmosphere. For conventional 12-well plate preparations, once the cells were confluent (approximately 80–90%), cells were passaged into 12 well plates. For the assays, cells in 75 cm^2^ flasks were trypsinised and seeded into a 12-well tissue culture plate (Sarstedt Ltd., Wexford, Ireland) at a density of 1 × 10^5^ cells/mL between passages 15–21 and cells were used once fully confluent (approximately 4 × 10^6^ cells/well at day 5–7). The media was changed every other day and supplemented with 2% FBS 24 h prior to use. For miniaturised 48-well plate preparations, the cells were grown and passaged as above. Cells were trypsinised and seeded into a 48-well tissue culture plate (Sarstedt Ltd., Wexford, Ireland) at a density of 1 × 10^5^ cells/mL between passages 15–21 and cells were used once fully confluent (approximately 2 × 10^6^ cells/well).

### 2.6. Exposure of Bacteria to Milk-Derived Powders

**Figure 1:**
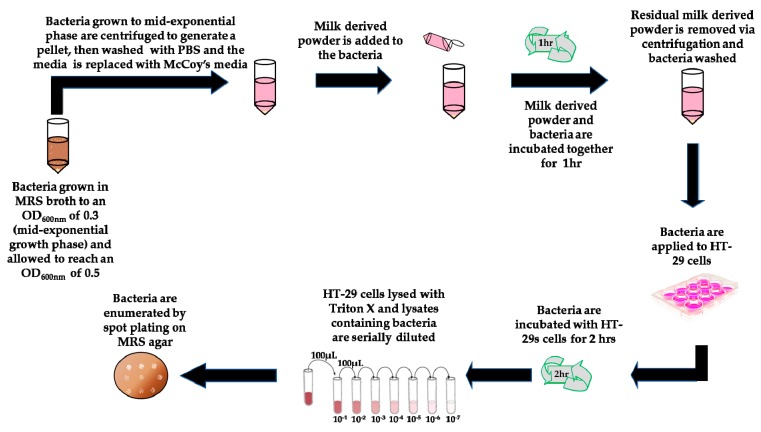
Schematic representation of *Bifidobacterium longum* subsp. *infantis* ATCC 15697exposure to milk-derived components and subsequent testing for adherence to HT-29 cells.

**Figure 2:**
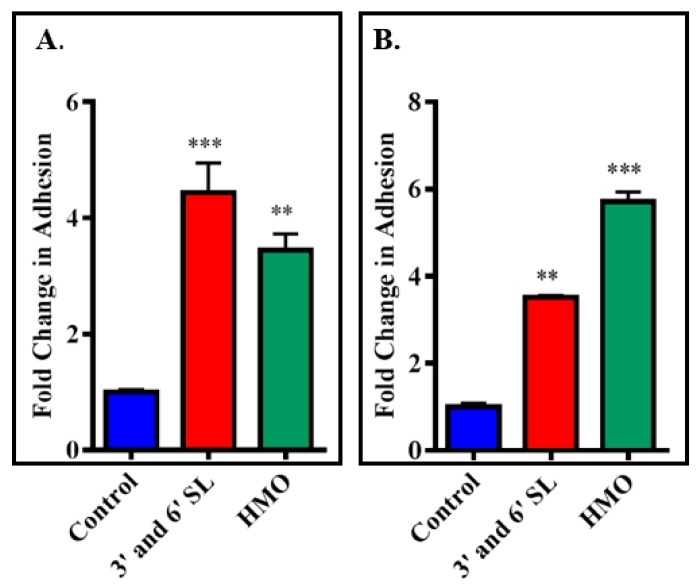
Comparison of adhesion of *B. longum* subsp. *infantis* ATCC 15697 to HT-29 cells following 1 h incubation with 5 mg/mL human milk oligosaccharides (HMO) and a mixture of 5 mg/mL 3′- and 6′-sialyllactose in a conventional adhesion assay (**A**) and miniaturised assay (**B**). Results are represented as percentage of adherent cells = [CFU/mL of recovered adherent bacteria ÷ CFU/mL of inoculum] × 100 and graphed as fold-change relative to percent adhesion of control, with error bars representing the standard deviation. (A) The unpaired non-parametric t-test was used and (B) Non-parametric one-way Anova multiple comparisons were used. ** *p*-value: <0.005, *** *p*-value: <0.002.

**Figure 3:**
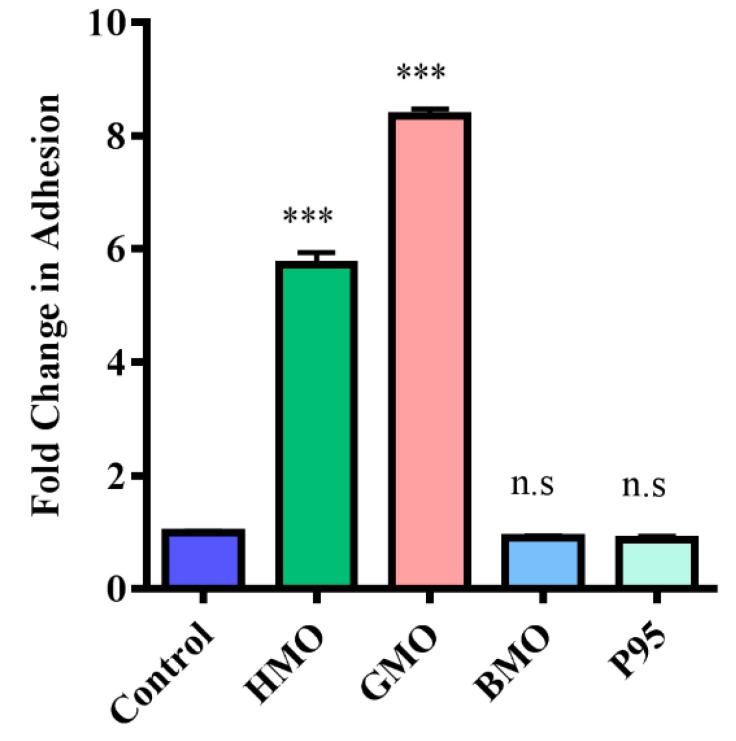
Adhesion of *B. longum* subsp. *infantis* ATCC 15697 to HT-29 cells following incubation with HMO, goat milk oligosaccharides (GMO), bovine milk oligosaccharides (BMO) and P95 using the miniaturised assay (represented as percentage of adherent cells = (CFU/mL of recovered adherent bacteria ÷ CFU/mL of inoculum) × 100). Results are graphed as fold-change relative to percent adhesion of control, with error bars representing standard deviation. The unpaired non-parametric *t*-test was used, *** *p*-value: <0.002, n.s: not significant.

**Figure 4:**
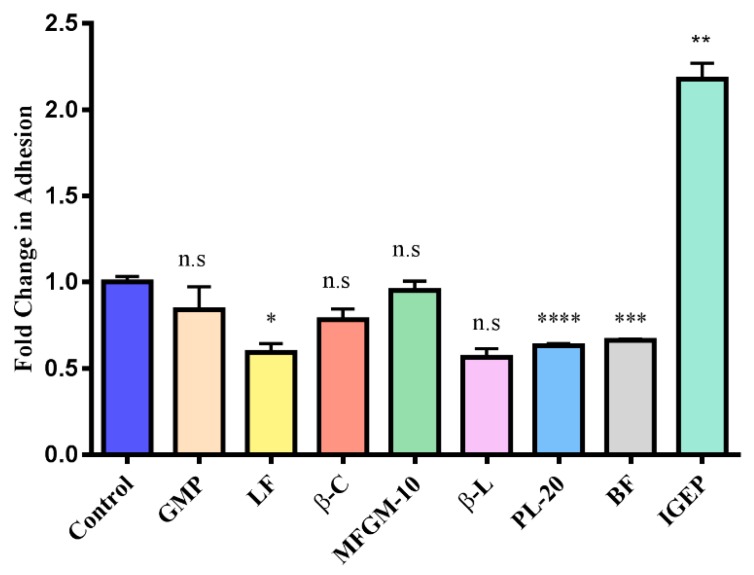
Adhesion of *B. longum* subsp. *infantis* ATCC 15697 to HT-29 cells following pre-treatment of bacteria with glycomacropeptide (GMP), lactoferrin (LF), β-casein (β-C), Lacprodan^®^ MFGM-10 (MFGM-10), β-lactoglobulin (β-L), Lacprodan^®^ PL-20 (PL-20), buttermilk fraction (BF) and immunoglobulin G enriched powder (IGEP) using miniaturised assay protocol (represented as percentage of adherent cells = [CFU/mL of recovered adherent bacteria ÷ CFU/mL of inoculum] × 100). Results are graphed as fold-change relative to percent adhesion of control, with error bars representing standard deviation. The unpaired non-parametric t-test was used, *: *p*-value: <0.05, ** *p*-value: <0.005, *** *p*-value: <0.002, **** *p*-value: <0.0001, n.s: not significant.

Exposure of the bacteria to milk-derived powders (Figure 1) was performed as previously described with minor modifications [9]. Bacteria were used at mid-exponential growth phase (18 h) and the OD_600nm_ was adjusted to 0.3 at the start of the assay, after which the cells were cultured for 1.5–2 h and used once an OD_600nm_ of 0.5 was reached (corresponding to approximately 1.6 × 10^8^ CFU/mL). Bacterial cells were washed twice with PBS by centrifugation. Cell pellets were re-suspended to a final OD_600nm_ of 0.5 in McCoy’s 5A tissue culture media supplemented with 2% FBS and either 5 mg/mL of HMO or a total of 5 mg/mL of a mixture of 3′ and 6′ SL at a ratio of 1:1 (after filtration using a 0.45 um filter, Sarstedt Ltd.). A non-supplemented negative control was also included, i.e., bacteria resuspended in McCoy’s 5A tissue culture media alone. Bacterial suspensions were then incubated for 1 h at 37 °C under anaerobic conditions. Following this, bacteria were harvested by centrifugation (3850× *g*, 5 min), the supernatants removed, and pellets were washed three times in PBS and then re-suspended in non-supplemented McCoy’s media prior to use in the adhesion assays. For the miniaturised bacterial assay exposures, *B. infantis* was cultured and prepared as above except cell pellets were re-suspended to a final OD_600nm_ of 0.25 in non-supplemented or supplemented McCoy’s 5A tissue culture media. A final concentration of 5 mg/mL of GMO, BMO, P95 and HMO was used plus a 5 mg/mL mixture of 3′ and 6′-SL at a ratio of 1:1. The results obtained for HMO and 3′ and 6′ SL were assessed and compared to ensure equivalence against the conventional assay (Figure 2). The same results were also included with the results obtained for the other oligosaccharides, GMO, BMO and P95 (Figure 3). A final concentration of 5 mg/mL of GMP, LF, β-C, MFGM-10, β-L, PL-20, BF or IGEP was also assessed in the miniaturised assay but was graphed separately (Figure 4).

### 2.7. Adhesion Assays

HT-29 cells were washed twice with PBS, and 500 µL of the bacteria and media suspensions were added to the wells, corresponding to approximately 40 bacterial cells per human cell in a 12-well plate. For the miniaturised assays, 250 µL of the bacteria and media suspensions were added to the wells, corresponding to approximately 40 bacterial cells per human cell, such that the ratio of bacteria to HT-29 cell was the same in the 12-well and 48-well assays. Bacterial cells were incubated with the HT-29 cells for 2 h at 37 °C under anaerobic conditions using an Anaerocult A system (Merck). The HT-29 cells were then washed five times with PBS to remove non-adherent bacteria. HT-29 cells were then lysed with 250 µL (for 48-well assay) or 500 µL (for 12-well assay) of 1% Triton^TM^ X-100 (Merck) for 5 min at 37 °C. The lysates were serially diluted and enumerated by spot-plating on MRS plates. The adhesion of the bacteria was determined as the percentage of original inoculum which attached, thus accounting for variations in the starting inoculum. Percentage adhesion = (CFU/mL of recovered adherent bacteria ÷ CFU/mL of inoculum) × 100. Overall, in this study the percentage of the original inoculum which adhered to the HT-29 cells was 0.49% ± 0.78%; this is similar to adhesion rates observed for this strain previously (0.40% ± 0.18%) by Kavanaugh et al. [9]. Adhesion assays were performed in triplicate on one occasion. Results were presented as the mean of three replicate experiments graphed as fold-change relative to percent adhesion of the control, with error bars of one standard deviation of the mean. All figures were generated using unpaired non-parametric t-test with the exception of Figure 2B where non-parametric one-way Anova multiple comparisons were used. Statistics were performed using GraphPad Prism version 7.00 for Windows (GraphPad Software, La Jolla, CA, USA, www.graphpad.com).

## 3. Results and Discussion

### 3.1. Comparison of Conventional and Miniaturised Assays

A miniaturised 48-well adhesion assay was compared with the conventional 12-well adhesion assay in an effort to reduce the volume of test sample consumption and increase the number of samples assayed together. Both the 48-well and 12-well assays demonstrated that pre-exposure of the strain to 5 mg/mL HMO, or a combination of 5 mg/mL 3′- and 6′-SL at a ratio of 1:1, resulted in increased adhesion to the HT-29 cells (Figure 2) consistent with previous work [8,9]. The 12-well assay resulted in a 3.4-fold increase in *B. infantis* adhesion after incubation with HMO, while the 48-well assay resulted in a 5.7-fold increase in adhesion. Similarly, incubation of *B. infantis* with 3′- and 6′-SL in the 12-well assay resulted in a 4.4-fold increase in adhesion, while the 48-well assay resulted in a 3.5-fold increase in adhesion which demonstrated the scalability of the method. Due to the inherent variability between adhesion assays, differences in the significance of adhesion rates between the conventional assay and the miniaturised assay are not unexpected. Thus, the miniaturised assay was comparable to, and as reproducible as, the conventional colonisation assay.

### 3.2. Effect of Milk Components on Growth of B. Longum subsp. Infantis ATCC 15697

*B. longum* subsp. *infantis* ATCC 15697 (*B. infantis*) was chosen for this study as it is a prototypical HMO consumer in the GI tract of the developing infant [16]. Genomic studies on the strain have demonstrated evolutionary adaptations for the utilisation of milk glycoconjugates [17] galacto-oligosaccharides (GOS) [18] and fructo-oligosaccharides (FOS) [19], along with HMO [10,16,18,20,21]. Consumption of such carbohydrates is facilitated by a number of glycosyl hydrolases and ABC transporters within the strain [16,17], making it a suitable model strain for this screening study. In this study, no increase in bacterial growth under adhesion assay conditions (1 h) was observed with the exception of supplementation with β-C, which resulted in a 47% (*p*-value: 0.053) increase in cell numbers, and β-L, which resulted in a 17% (*p*-value: 0.037) decrease in bacterial cell numbers (Table S1). In whole bovine milk, there are four major forms of casein, αs1-, αs2-, β- and κ-caseins which make up 38%, 10%, 35% and 12%, respectively [22]. Some of these forms and fractions of these forms have been demonstrated to have growth-promoting activities [23,24]. Bovine β-L has been shown to have anti-microbial effects against mastitis-causing bacteria [25]. This anti-microbial effect could help explain the decrease in *B. infantis* numbers observed during the 1 h incubation period in the presence of β-L.

### 3.3. Screening Milk Components for Increased Adhesion of B. longum subsp. Infantis ATCC 15697 to HT-29 Cells

In order to identify milk components capable of modulating the adhesion of commensal strains using miniaturised adhesion assays, *B. infantis* was incubated separately with oligosaccharides isolated from different sources including human (HMO), goat (GMO), bovine (BMO), and a commercial prebiotic (Beneo Orafti P95) which acted as a negative control [9], for 1 h at 5 mg/mL, after which the strain’s ability to adhere to HT-29 was determined. This concentration was selected based on physiological concentrations of oligosaccharides present in human milk. Bacterial adhesion to HT-29 cells following pre-treatment of the bacteria with GMO resulted in a marked increase in adhesive ability of the strain (Figure 3), as represented by an 8.3-fold increase in adhesion versus the control.

Previously, *B. infantis* was shown to have improved adherence following incubation with HMO using in vitro colonisation models [8,9]. Kavanaugh et al. [9] reported a transcriptional response in the presence of three different oligosaccharide treatments (3′-SL, 6′-SL and 3′- and 6′-SL combined) and an overall up-regulation of genes involved in adhesion with a down-regulation of genes involved in complex oligosaccharide metabolism. However, while that study focused on only HMO and combinations of 3′- and 6′-SL, the current study is the first to examine the effect of oligosaccharides and other milk-derived components isolated from domestic animal milks on the adhesive properties of *B. infantis*, thereby potentially overcoming the limitation in the availability of many HMOs. Each oligosaccharide powder used in this study contained 3′ and 6′ SL (Table 2). In this study, bacterial cells were incubated with the HT-29 cells for 2 h at 37 °C under anaerobic conditions using an Anaerocult A system (Merck, Dannstadt, Germany). While anaerobic conditions are not optimal for HT-29 cells, previous studies have demonstrated that minimal negative effects occur within this time period while allowing for optimal conditions for the bacteria [9].

Previous studies have shown that goat milk and human milk share many common oligosaccharide structures [26] such as β3′-galactosyllactose, β6′-galactosyllactose, 2′-fucosyllactose, lactose-*N*-hexaose, 6′-*N*-acetylneuraminyllactose and 3′-*N*-acetylneuraminyllactose. In fact, the overall oligosaccharide profile of goat milk is more similar to human milk than either bovine or ovine milk [27]. Added to that, although goat milk has a substantially lower concentration of oligosaccharides (0.25–0.30 g/L) when compared to human milk (3–20 g/L), it contains 5 to 8 times more oligosaccharides than bovine milk (0.03–0.06 g/L) and 10 times more than ovine milk (0.02–0.04 g/L), [27,28]. Interestingly, GMOs have previously been shown to have other positive biological effects. For instance, GMOs can modulate the immune response [29,30] and have also been shown to promote the growth of probiotic bacteria [31]. The current study further highlights their potential for inclusion in formulations where breastfeeding is not possible. Currently, many formulations based on goat milk are on the market, and methods for the large-scale isolation of GMOs have been investigated, which may allow future clinical trials [32].

In contrast to the other oligosaccharide powders under investigation, pre-treatment with P95 (0.12-fold decrease) or BMO (0.1-fold increase) did not result in a statistically significant change in adhesion of *B. infantis* to the intestinal cells (Figure 4). P95 had no effect on *B. infantis* adhesion in the study performed by Kavanaugh et al. [9], therefore the result obtained in this study is not surprising. The BMO powder, however, contains high quantities of 3′- and 6′-SL (Table 2) with minimal lactose and therefore may be expected to positively influence the adhesion of the strain. This result suggests that in contrast to pure 3′- and 6′-SL, which seem to have a synergistic effect on adhesion [9], the combination of multiple oligosaccharides within the BMO pool had no effect on the adhesion capacity of the strain and may actually inhibit the activity of 3′- and 6′-SL in promoting adhesion.

In this study, apart from free oligosaccharides, IGEP, which is an IgG enriched powder from bovine whey, also enhanced adhesion of *B. infantis* to intestinal cells by 3.3-fold (Figure 4). Numerous studies have highlighted IgG’s bioactivity, specifically its anti-infective capabilities [33,34]. IgG binds to many gastrointestinal pathogens including *Shigella flexneri*, *Escherichia coli*, *Clostridium difficile*, *Streptococcus mutants*, *Cryptosporidium parvum*, *Helicobacter pylori*, and rotavirus [35]. Indeed, the main role of milk IgG is to agglutinate bacteria, neutralise toxins, inactivate viruses and provide an environment favourable for the growth of beneficial bacteria [34,36]. Considering the immunomodulatory and bacterial binding properties of IgG, IGEP, which is particularly rich in IgG, may promote adhesion of *B. infantis* by acting as a bridging molecule through interacting with both the mammalian and bacterial cells simultaneously. Alternatively, *B. infantis* is known to encode an endoglycosidase, EndoBI-1 (glycosyl hydrolase family 18), which cleaves many major types of N-linked oligosaccharides found on glycoproteins and could thus release intact oligosaccharides from immunoglobulin [37], thereby providing free oligosaccharides which may function similarly to HMO. However, further studies are required to investigate whether this is a possibility.

Statistically significant reductions in bifidobacterial adhesion were observed with exposure to LF, which resulted a 40% reduction in adhesion (*p*-value: 0.0151), the butter milk fraction, which resulted in a 34% reduction (*p*-value: 0.0002), and PL-20, which resulted in a 37% decrease (*p*-value: <0.0001) (Figure 4). As mentioned, Kavanaugh et al. [9] reported an up-regulation in genes associated with adhesion, and a down-regulation in genes associated with oligosaccharide consumption following a 3 h pre-incubation period with 3′- and 6′-SL. The components resulting in reduced adhesion of the strain following the 1 h pre-incubation period could potentially cause *B. infantis* to preferentially up-regulate genes involved in carbohydrate metabolism, rather than use energy on adhesion activities. However, no increase in growth was observed within the 1 h incubation period in this work with the exception of β-C (Table S1). LF is known to have prebiotic abilities, promoting the growth of various lactobacilli [38], and antimicrobial activity [39,40], and also displays anti-adhesive effects against pathogens [41,42]. Further investigations are required to explain the decrease in adhesion observed in the current study.

The buttermilk fraction and PL-20 are rich in glycoproteins and are associated with many bioactive properties. Previously, milk fat globule membrane glycoproteins were shown to have anti-adhesive properties against a range of pathogens including rotavirus and various enteric bacteria [12,36,43,44]. Again, given the carbohydrate-rich nature of constituent glycoproteins, the bacteria may favour carbohydrate consumption rather than adhesion when incubated with the buttermilk fraction and PL-20. This however has yet to be confirmed, as significant increases in growth were not observed in the 1 h pre-treatment period (Table S1). Another consideration is that commensal strains often adhere to cells using the same host ligands as employed by pathogens [9,45]. As many of the milk components used in this study which resulted in reduced adhesion of the bifidobacterial strain are also anti-adhesive against pathogens, it may be that they are competing with the colonisation factors expressed by *B. infantis* to colonise the host.

In this study, high molecular weight compounds, such as lactoferrin, β-lactoglobulin and the buttermilk fraction resulted in a decrease in bifidobacterial adhesion. However, it is important to note that under conditions, these would likely be digested by the host prior to reaching the colon and therefore could potentially confer different biological effects to those observed in vitro. In contrast, the oligosaccharides tested in this study resulted in an increase in adhesion. Milk oligosaccharides are known to resist digestion, and thus can reach the colon where their presence can influence the development of a healthy gut microbiota. While IGEP is a high molecular weight protein that can be partially digested by the host, the resulting glycopeptides may still have bioactive effects on adhesion.

## 4. Conclusions

Considering the implications of human-derived products such as HMO and 3′- and 6′-SL, inclusion of GMO as a food ingredient in infant formulations to boost numbers of bifidobacteria could be a viable alternative, particularly considering the growing popularity of formulas based on goat milk. Bovine IGEP is relatively easy to isolate and is currently being produced by dairy companies in Europe for use as a food ingredient. In addition, while this research indicates the potential effects that milk-derived components may have on the gut microbiota, more supporting data are required. Future studies to investigate the effects that these powders may have on the colonisation of other gut- associated commensal and pathogenic bacteria are necessary. This study provides insight into the role milk components may have modulating commensal transient adhesion, as well as highlighting alternatives to HMO and 3′- and 6′-SL which are more commercially attractive to produce on a large scale. In addition, these results demonstrated that miniaturised assays are a feasible alternative to conventional assays where the yield of bioactive compounds is too low to support a conventional study. As the plate counting method is time-consuming and can be variable, fluorescent-labeling of the bacteria to measure the bacteria adhering using a fluorimeter and/or qPCR may improve the efficiency and yield of the miniaturised assay.

## Figures and Tables

**Table 1:** List of milk-derived components used in study.

Powder	Abbreviation	Source
Goat milk oligosaccharides	GMO	Isolated in-house
Bovine milk oligosaccharides	BMO	Isolated in-house
Human milk oligosaccharides	HMO	Isolated in-house
3′-sialyllactose and 6′-sialyllactose	3′ and 6′ SL	Merck
Glycomacropeptide	GMP	Agropur foods international
Lactoferrin	LF	Upfront Chromatography A/S
β-casein	β-C	Merck
Lacprodan^®^ MFGM-10	MFGM-10	Arla food ingredients
β-lactoglobulin	β-L	Merck
Lacprodan^®^ PL-20	PL-20	Arla food ingredients
Butter milk fraction	BF	Isolated in-house
P95	P95	Beneo Orafti
Immunoglobulin G Enriched Powder	IGEP	Upfront Chromatography A/S

**Table 2:** Level of 3′ and 6′ sialyllactose present in the isolated oligosaccharide pools.

Powder	3′ Sialyllactose (μg/mL)	6′ Sialyllactose (μg/mL)
GMO	37.14	35.28
HMO	46.90	59.00
BMO	48.22	26.79

## Supplementary Materials

The following are available online at http://www.mdpi.com/2304-8158/7/12/196/s1, Table S1: Significant differences in growth of *B. infantis* following 1 h incubation with the milk derived components.
