# Supplementary material for: Mining Milk for Factors which Increase the Adherence of Bifidobacterium longum subsp. infantis to Intestinal Cells
Source: Foods. 2018 Dec 3;7(12):196. doi: 10.3390/foods7120196 (PMC6306836; doi:10.3390/foods7120196)
Supplement: Supplementary file 1 [file foods-07-00196-s001.pdf]

Supplementary Information

Table SI: Significant differences in growth of *B. infantis* following 1 hour incubation with the milk derived components.

Powder†	Percentage Difference	Significance
**β-C**	65%	0.053*
**β-L**	-7%	0.037*

*Results for individual powders are represented as the average of three experimental replicates.

† HMO, GMO, BMO, 3' & 6' SL P95, GMP, LF, MFGM-10, PL-20, BF and IGEP did not result in a significant difference in growth of *B. infantis* following 1 hour incubation
